# The Influence of Formation Speed in Centrifugal Slip-Casting Method on the Microstructure of Al_2_O_3_-Cu-Cr Gradient Composites

**DOI:** 10.3390/ma16196501

**Published:** 2023-09-30

**Authors:** Justyna Zygmuntowicz, Aneta Dwojak, Paulina Piotrkiewicz, Marcin Wachowski, Waldemar Kaszuwara

**Affiliations:** 1 Faculty of Materials Science and Engineering, Warsaw University of Technology, 141 Wołoska Str., 02-507 Warsaw, Poland; an.dwojak@gmail.com (A.D.); paulina.piotrkiewicz.dokt@pw.edu.pl (P.P.); waldemar.kaszuwara@pw.edu.pl (W.K.); 2 Faculty of Mechanical Engineering, Military University of Technology, Gen. S. Kaliskiego 2 Str., 00-908 Warsaw, Poland; marcin.wachowski@wat.edu.pl

**Keywords:** functionally gradient materials, hybrid composites, ceramic–metal composites, centrifugal slip casting

## Abstract

This work focuses on the production of gradient composite materials with an alumina matrix containing copper and chromium and examines the effect of the reinforcement and casting speed on the obtained microstructure. Al_2_O_3_-Cu-Cr composites with a microstructure gradient were produced via centrifugal slip casting. The research reveals that adding chromium to the Al_2_O_3_-Cu system improves the connection between the ceramic and metal particles, probably by reducing the contact angle at the interface between the ceramic and metallic phases during sintering. Additionally, it was found that higher casting speed was conducive to obtaining a better connection at the interface of ceramics and metal.

## 1. Introduction

Al_2_O_3_ matrix composites exhibit high-temperature resistance, thermal shock resistance, excellent abrasion resistance, and corrosion resistance. Reinforcement with copper allows obtaining a material with good electrical and thermal conductivity and improves fracture toughness. The combination of the properties described above allows for an attractive material in many applications, i.e., the production of sensors, heat sensors, transmission pipes, and structural elements. Nevertheless, it should be remembered that the properties of the produced composite depend on the amount of reinforcement introduced into the matrix, raw materials, additions of other metals, and the technology and production parameters.

The technology most frequently described in the literature for producing Al_2_O_3_-Cu composites is the hot-pressing (HP) technique [1,2,3,4,5]. An example of an Al_2_O_3_-Cu composite produced by this method is the material described by Niihara [1] containing 5%_vol_ of copper. The composite obtained from a mixture of Al_2_O_3_-Cu powders and sintered at a temperature of 1600 °C reached a relative density of ρ = 97 ± 1% and a fracture toughness coefficient K_IC_ = 4.72 ± 0.20 MPa·m^1/2^. Slightly better properties were obtained for the composite made of a mixture of Al_2_O_3_/CuO powders (ρ = 98 ± 1, K_IC_ = 5.40 ± 1.15 MPa·m^1/2^) [3]. The properties of Al_2_O_3_/Cu composites obtained by infiltration of a ceramic preform with an open porosity of 15%_vol_ were described by Travitzky [6]. According to his results, the composites produced had excellent strength properties: K_IC_ = 6.70 ± 0.25 MPa·m^1/2^ and a microhardness of 8.30 ± 0.24 GPa. Good relative density of 99.1% and strength properties were also achieved for the composite produced in the process of sintering a mixture of Al_2_O_3_/Cu powders using the spark plasma sintering (SPS) technique [7,8]. According to the conducted research [9] on Al_2_O_3_/Cu composites produced by slip casting with a content of 1–10%_vol_ of Cu, lower density was observed than in the case of the methods described above. In addition, the optimal combination of impact strength and microhardness properties was obtained for composites with a Cu content below 5%_vol_. Higher Cu content decreased both microhardness and impact strength, which most likely resulted from lower dispersion of the metallic phase in the composite volume. At the same time, observations of the fracture surfaces of composites confirmed the effect of strengthening the ceramics with metal particles. The centrifugal casting technique is promising for producing Al_2_O_3_-Cu composites [10]. According to the data presented in the literature, this technique makes it possible to create a gradient of the distribution of the metallic phase in the matrix. The obtained results of microhardness distribution on the cross-section of the composite show that they depend on the concentration of the metallic phase in a given area of the sample and range from 129 HV1 for the area with the most significant copper agglomeration to 2020 HV1 for the area where no metallic phase was observed. Moreover, the centrifugal slip-casting method is an innovative process that enables obtaining finished materials in the shape of a sleeve [10]. Analogized to other forming processes, the centrifugal slip casting main benefit is obtaining a finished product as a pipe. The centrifugal slip-casting process does not use large amounts of organic substances as binders. A large amount of binders can result in problems with its removal during the sintering process and give the prospect of defects appearing during the removal of the binder in the products obtained, e.g., by hot pressing or the injection technique [10].

Difficulties that may be encountered during the production of the Al_2_O_3_-Cu composite are the formation of high lattice stresses as a result of differences in thermal expansion of ceramics and metal, weakening the strength of the material, as well as poor wettability of ceramics by metal [11,12], leading to the phenomenon of copper flowing out of the matrix. The wettability between the components can be improved by handling the newly formed components, creating intermediate phases that melt and then wet the components or react with the metal and lower its surface tension. Another way is to introduce an oxide into the sintered system, which will improve wettability and then be reduced at the sintering temperature to a high-melting metal [13]. Better wettability can be achieved by adding other metals that exhibit good wettability of ceramics [14,15]. 

This work focuses on the production of gradient composite materials with an alumina matrix containing copper and chromium, as well as examining the effect of the reinforcement and casting speed on the obtained microstructure of the material. The scope of work includes: characterization of the starting powders used for the production of ceramic–metal composites, production of Al_2_O_3_-Cu-Cr composites with the content of 2.5%, 5%, 10%, and 15%_vol_ of the metallic phase by centrifugal casting using two variants of the casting speed, macroscopic assessment of the quality of the produced composites, examination of the physical properties of composites, i.e., relative density, open porosity, and water absorption by the Archimedes method, microscopic assessment of the quality of sinters and the obtained gradient of the structure on the cross-section using an optical microscope, SEM (scanning electron microscope) observations in order to assess the connection at the ceramic–metal interface and the influence of chromium content on improving the wetting of Al_2_O_3_ by copper, and analysis of the chemical composition of energy-dispersive (EDS) stereological studies of the size of metallic phase agglomerates in individual zones of the cross-section composites.

## 2. Materials and Methods

### 2.1. Research Materials

The starting materials for forming ceramic–metal composites included alumina powder under the trade name of TM-DAR from Tamei Chemicals Co. (Tokyo, Japan) and copper and chromium powders by Createc (Kraków, Poland). Powder specifications are shown in Table 1. The choice of powders was dictated by their high purity. Moreover, Al_2_O_3_ powder (TM-DAR) is used by a lot of scientists all over the world [16,17,18].

### 2.2. Phase Composition Analysis

The analysis of the phase composition of the powders used in the experiment was carried out by X-ray diffraction. The measurement was performed using a Rigaku Mini Flex II diffractometer (Tokyo, Japan). The radiation source was an X-ray tube with a copper anode with a wavelength of the Kα radiation line of λ = 1.54178 Å. The test was carried out at angles θ 20–100°, using a voltage of 30 kV and a current of 15 mA. The goniometer rotation speed was 0.02°/min, and the diffraction signal counting time was 3 s.

### 2.3. Laser Analysis of Powder Particle Size Distribution

The particle size distribution (PSD) of the starting powders was determined by dynamic light scattering (DLS). The measurement was carried out using the HORIBA LA—950 analyzer. (London, ON, Canada) The light source used was a red laser (with a wavelength of 630 nm) and a blue laser (with a wavelength of 460 nm), which allowed us to obtain a wide measurement range of particle size of 0.01–3000 µm. Alumina powder was tested in an aqueous environment, while isopropanol was used as a dispersant for metal powders.

Based on the obtained results, the homogeneity of the powders, the diameters of the particles below which their probability of occurrence is 10 and 90% (D_10_ and D_90_), and the average particle sizes of the powders were determined.

### 2.4. Examination of the Actual Density of Starting Powders

The density of the starting materials used to prepare the samples was measured using an AccuPyc 1340 II helium pycnometer (Micromeritics Instrument Corporation, Norcross, GA, USA). The test powder was placed in a measuring cell and weighed. The powder mass value was entered into the pycnometer, which measured the powder volume. The volume was determined based on the differences in the volume of helium introduced into the cell filled with powder and the empty cell in each cycle. Gas was introduced into the vessel at a pressure of 19.5 psig. The measurement ended when the values of the determined densities (ρ (g/cm^3^)) stabilized in subsequent cycles. For alumina and copper powder, it was 300 cycles, and for chromium, it was 400 cycles.

### 2.5. Determination of the Specific Surface Area of Powders Using the BET Method

The size of the total surface area, i.e., the ratio of the total surface area available for gas adsorption to the powder mass, was determined based on the analysis of Brunauer–Emmett–Teller (BET) adsorption isotherms. The measurement of physisorption isotherms on the powder surfaces at the temperature of liquid nitrogen, and the adsorbate used was nitrogen. The study was performed using the ASAP2020 apparatus (Micrometics Instruments, Micromeritics Instrument Corporation, Norcross, GA, USA). Before the measurement, the samples were degassed under reduced pressure at 90° for 1 h and then at 300 °C for 4 h. SPET-specific surfaces (m^2^/g) were determined based on five points of the recorded physisorption isotherm in the range of relative pressures of ρ/ρ_0_ = 0.5–0.3 for Al_2_O_3_ powder and ρ/ρ_0_ = 0.01–0.1 for Cu and Cr powders. The values of S_BET_ and ρ were used to determine the average particle size of the powder d_BET_.

### 2.6. Microscopic Observations of Powders Using an SEM Microscope

Observations of the morphology of the powder particles were carried out using a JSM-6610 scanning electron microscope. The secondary electron detector—SE (Secondary Electrons) mode and the voltage of 20 kV were used in the research.

### 2.7. Archimedes Method

The masses of the samples of the composites produced after sintering m and the masses of the samples obtained during hydrostatic weighing (Archimedes method) were used to determine the selected physical properties. Calculated theoretical densities of the produced composites depending on the content of the metallic phase based on the rule of mixtures have been shown in Table 2.

### 2.8. Sinter Microstructure Observations

To observe the microstructure of the composites, cross-sections of sinters were made by incorporating them in a conductive resin and grinding the surface on sandpaper with a gradation of 240, 400, 600, 800, 1200. and then polishing with a diamond suspension with a particle size of 3 µm and 1µm. The prepared microsections were observed using an AxioVert 40MAT optical microscope. To illustrate the microstructure, cross-sectional panoramas were made, which were then used to observe the distribution of the metallic phase and assess porosity and the occurrence of microcracks.

Observations of the microstructure of the cross-sections of the composites were carried out using the JSM-6610 scanning electron microscope. The observations were carried out in the SE and backscattered electrons (BSE) modes, using a voltage of 15 kV. The observations were used to evaluate the bonding at the ceramic–metal phase interface.

### 2.9. Chemical Composition Analysis

The analysis of the chemical composition using the EDS detector was carried out during the observation on the SEM microscope of the JSM-6610 type. The analysis was point-based. The purpose of the analysis was to identify the observed areas.

### 2.10. Image Analysis

Image analysis of the metallic phase agglomerates’ size in each gradient zone was carried out using the MicroMeter program [19,20,21]. This program allows you to determine the diameters of particles based on the analysis of the microstructure image of the tested material [20,21]. The average particle size calculated by the program is an equivalent diameter—corresponding to the diameter of a circle whose surface corresponds to the surface of the analyzed particle [19,20,21,22]. For the analysis of each zone, 10 images of the microstructure of a given sample were used, made using the SEM TM1000 microscope at 300× magnification. The data obtained during the analysis were used to develop histograms of the size of the metallic phase areas and their average sizes in each zone. Based on the data on the area of the metallic phase areas and the area of the analyzed range, the shares of the metallic phase in each gradient zone were determined.

### 2.11. Molding Process

The production of ceramic–metal composites consisted of three successive technological processes: centrifugal casting, drying, and sintering.

The first stage of the centrifugal casting process was the preparation of the slurry. A 30 cm^3^ slurry with 50%vol of the solid phase was prepared for each process. The share of the metallic phase for individual slurries was 2.5%, 5%, 10%, and 15%_vol_ of the total content of the solid phase in the slurry. The dispersing phase of the powder particles was distilled water with the addition of fluidizers: citric acid (CA) and di-ammonium hydrogen citrate (DAC). The components of the mass were mixed in a high-speed planetary mixer of the THINKY MIXER ARE-250 type for 8 min at the speed of 1000 rpm to homogenize the resulting mass, and then it was deaerated for 2 min at the speed of 2000 rpm. Centrifugal casting was carried out in plaster molds in the shape of thick-walled sleeves. The casting process for each variant of the content of the metallic phase was carried out at the following speeds: 3000 and 1500 rpm for 120 min.

In total, 8 samples with different content of the metallic phase were produced in two variants of the casting speed (Table 3).

The cast fittings were dried in the mold using a vacuum dryer at 40 °C for 24 h. The purpose of the drying process was to remove excess moisture.

Dried samples were placed in a corundum “boat,” covered with alumina powder, and then sintered. The samples were heated to the sintering temperature at the speed of 0.2 °C/min in the range of 0–120 °C, then at the speed of 1°/min to the temperature of 750 °C and in the last stage at the speed of 2°/min. Sintering was carried out at 1400 °C for 2 h, then cooled at a rate of 4°/min. The process was carried out in a reducing atmosphere containing 95% Ar and 5% H_2_.

## 3. Results

In the first stage of the research, the initial powders used to produce the composites were characterized in terms of particle size, shape distribution, and chemical composition.

Phase analysis confirmed the composition of the starting powders used to produce the Al_2_O_3_-Cu-Cr composites. The diffraction patterns obtained during the analysis show only the peaks from the starting materials, which confirms their high purity.

**Figure 1:**
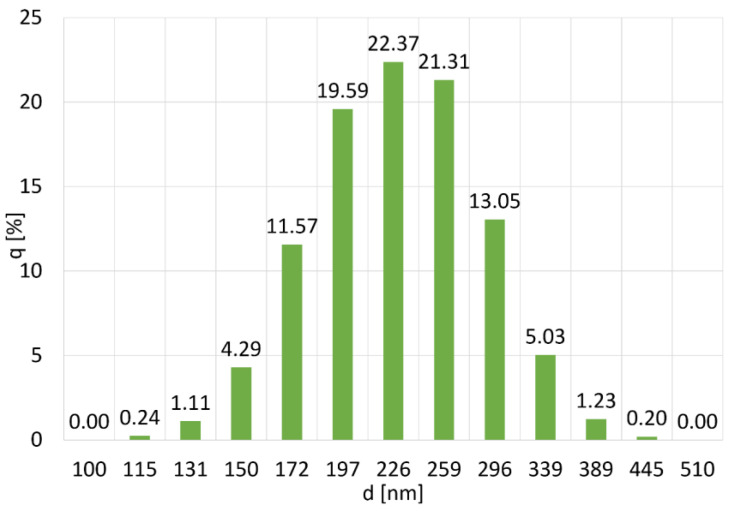
Particle size distribution of Al_2_O_3_ powder, where (%) is the number of particles with diameter d.

**Figure 2:**
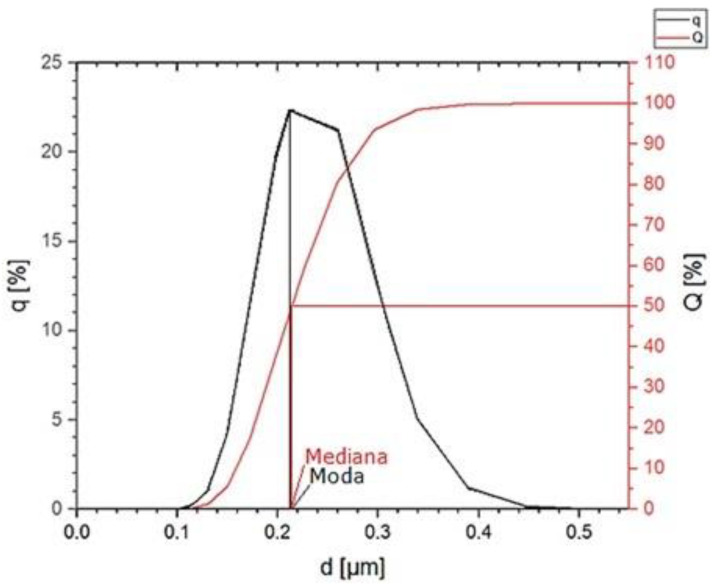
Particle size distribution q (%) and cumulative particle size distribution Q (%) of the Al_2_O_3_ powder.

The PSD distribution of the Al_2_O_3_ powder is symmetrical (Figure 1), proving the powder’s high homogeneity. This is also evidenced by the value of the mean being close to the value of the mode and the median (Figure 2). The analysis showed that the average particle size is 218 ± 49 nm. The most numerous fractions are particles with a diameter of 226 nm and 259 nm, the largest in the population. The probability of occurrence of particles with a diameter below 158 nm is 10%. The measured particle sizes are approximately twice the manufacturer’s specifications. This may be due to the formation of powder agglomerates. The tendency of the powder to form agglomerates will be determined later in the work based on SEM observations.

**Figure 3:**
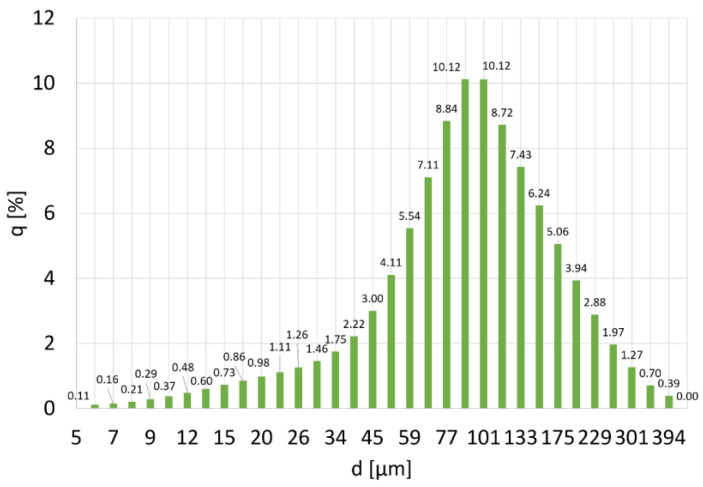
PSD distribution for copper powder, where q (%) is the number of particles with diameter d.

**Figure 4:**
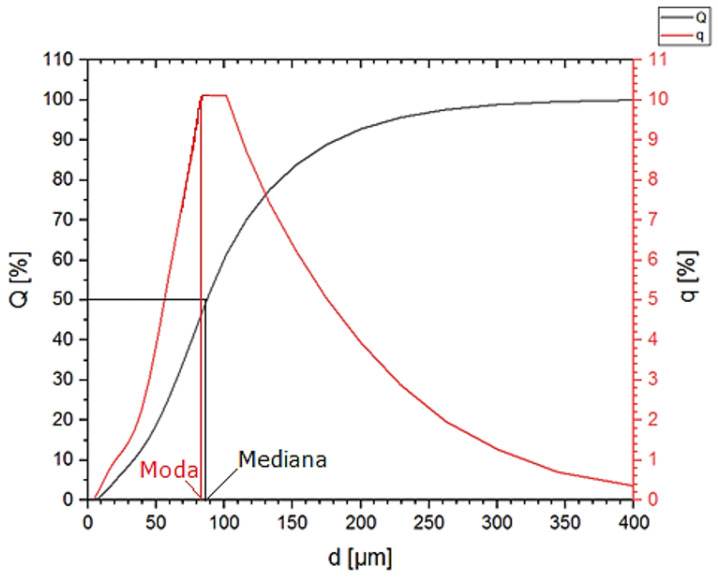
Particle size distribution q (%) and cumulative particle size distribution Q (%) of Cu powder.

The analysis of the size distribution of copper particles showed that the most numerous fractions are particles with a size of 101 and 89 µm. Their share in the powder volume is 10% for each fraction (Figure 3). The average particle size is 100 ± 61 µm. The mode value is 83 µm, and the median is 87 µm (Figure 4). The particle size distribution is vast, but the powder can be considered homogenous due to the small fraction of the smallest particles. The D_10_ value for Cu powder is 33 µm and D_90_ = 182 µm.

**Figure 5:**
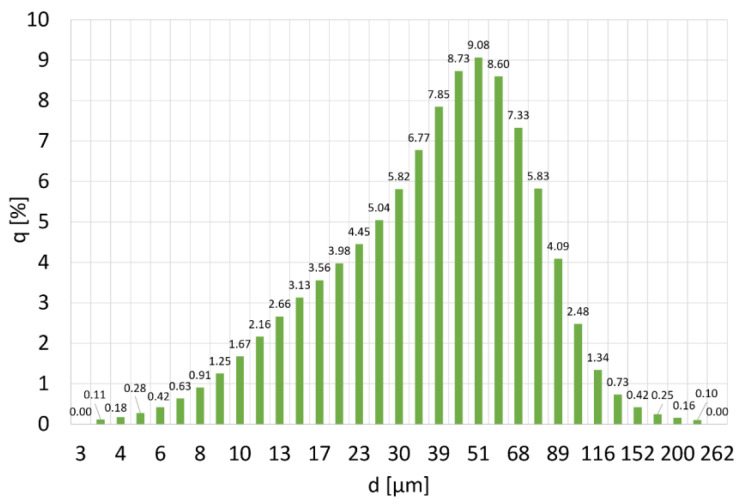
PSD distribution of chromium powder, where q (%) is the amount of particles with diameter d.

**Figure 6:**
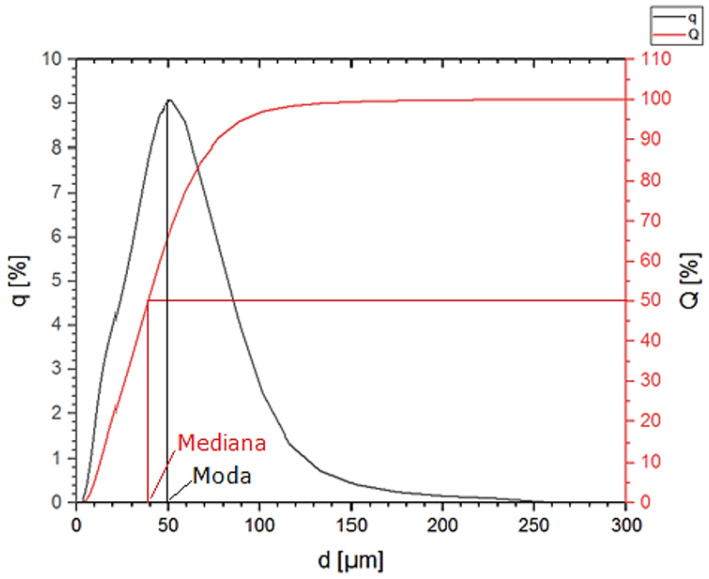
Particle size distribution q (%) and cumulative particle size distribution Q (%) of Cr powder.

According to the PSD distribution for chromium powder obtained in the DLS analysis, the most numerous fractions in the population are particles with a size of 45 µm, 51 µm, and 59 µm. Their content in the population is approx. 9% (Figure 5). The particle size distribution is wide. The determined values of the mode and the median (Figure 6) are close to the mean diameter of the particles. However, it is the least homogenous of the composites used to produce it. The average particle size of the population is 43 ± 27 µm. The probability of occurrence of particles below 13 µm in diameter is 10%, and particles with a diameter of less than 77 µm are 90%.

The diameters of powder particles calculated using the densities obtained in pycnometric measurements and specific surfaces determined based on the BET absorption isotherm differ from the diameters provided by their manufacturers. These differences are substantial in the case of metal powders. The d_BET_ values (Table 4) of Cu and Cr powders are significantly lower than their specifications. This is due to the irregular shape of the particles and the developed surface (calculations using the BET isotherm are carried out assuming that the particles are spherical).

**Figure 7:**
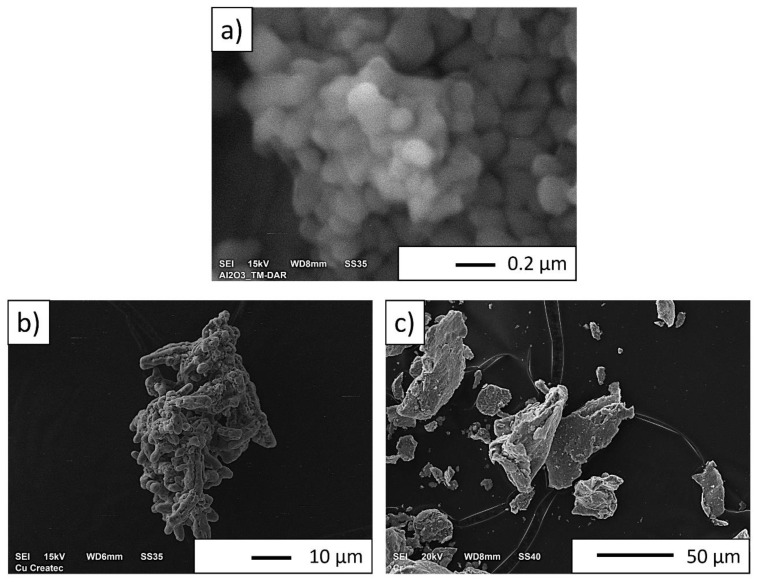
Powders used to form composites: (**a**) Al_2_O_3_; (**b**) Cu; (**c**) Cr.

Microscopic observations of the Al_2_O_3_ powder revealed a high uniformity of particle size and shape (Figure 7a). The particles have a regular shape with smooth edges. Their size is approx. 0.25 µm. Observations revealed the tendency of the powder to form agglomerates.

Copper powder particles have a dendritic shape with irregular morphology (Figure 7b)

Based on the observations using the SEM microscope, it was found that the Cr powder is characterized by flake morphology (Figure 7c). Microscopic observations confirmed the powder particle size determined in the DLS study.

After the molding process, macroscopic observations of the samples showed no visible pores, delaminations, or cracks on the external surfaces and their cross-sections. The centrifugal slip-casting method allowed us to obtain a structure with a gradient of the distribution of metallic particles.

In the first stage of the study of sintered composites, the selected physical parameters of the samples were determined. The relative density of A series composites ranges from 80.35 ± 0.43% to 95.7 ± 0.48%. Sample A_15 has the lowest relative density in this series. This value is much lower than the density of other composites, which may be due to numerous cracks that lowered the density of this composite and to the outflow of a significant amount of the liquid phase during the sintering process. It was found that the highest density was obtained for the A_10 composite. The densities of A_2.5 and A_5 composites are similar. Based on the obtained results, it was noticed that the relative density increases with the increase in the proportion of the metallic phase to the content of 10% volume.

On the other hand, for Series B, the obtained relative densities range from 93.68 ± 1.09% to 97.74 ± 0.31%. Sample B_10 has the lowest density and B_2.5 the highest. A high relative density characterizes all produced composites, and a lower casting speed was conducive to obtaining a higher density. This conclusion is surprising but well-confirmed experimentally. Using a higher spinning speed during the centrifugal slip-casting process leads to a greater concentration of Cu particles. As a result, in areas rich in Cu, sintering in the solid phase (Al_2_O_3_) is difficult. In addition, large Cu concentrations lead to locally high stresses. Large Cu concentrations in the sample may cause the measured density and shrinkage to be lower. Thus, high open porosity and water absorption are a consequence of this.

The determined open porosity of the A series composites ranges from 0.63 ± 0.12% for the A_5 composite to 9.62 ± 1.92% for the A_15 composite. The high porosity of the A_15 sample most likely results from numerous cracks. Sample A_2.5 has a much higher porosity than the other composites from both series, for which P_o_ = 4.11 ± 1.49% (Table 5). The open porosity ranges from 0.21 ± 0.07% for composites cast at lower speeds to 0.77 ± 0.12%. The most minor open porosity is found in Sample B_2.5, and the highest B_15. Thus, the open porosity increases with the increase in the proportion of the metallic phase, which may indicate poor wettability of the ceramics by the liquid metallic phase during sintering. Composites cast at a higher speed have a higher open porosity than those cast at a slower speed and with the exact content of the metallic phase. The Cu particles are more concentrated at a higher speed during the molding process. A more significant amount of copper agglomerates was found in the fittings produced at a higher casting rate, which may cause more effortless Cu flow from the sample during sintering, which explains the density of the obtained A_15 samples.

Water absorption determined for Series A composites ranges from 0.16 ± 0.03% to 2.62 ± 0.53%. The lowest water absorption, which proves the lowest porosity, has composite A_5, slightly lower than the water absorption of composite A_10. The A_15 composite has the highest porosity, which results from many cracks, similar to the low ρw and high Po of this composite. The determined absorbability of the B series composites ranges from 0.05 ± 0.02% obtained for the B_2.5 composite to 0.18 ± 0.03% obtained for the B_15% composite. The porosity of the B series composites increases with the increase in the proportion of the metallic phase. Composites cast at slower speeds have lower porosity.

**Figure 8:**
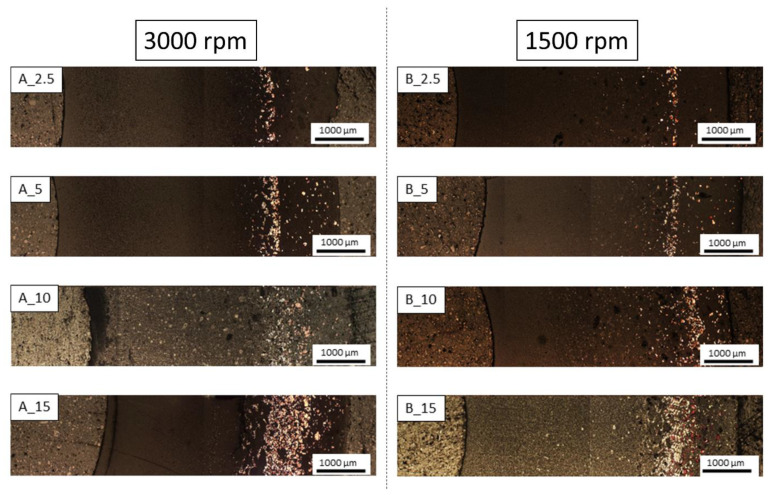
The observation of the microstructure of the composite using a light microscope.

Based on the observation of the microstructure using a light microscope, it was found that a gradient of metallic phase distribution was formed on the cross-sections of the composites (Figure 8). In the areas at the inner edge of the composites of both series, i.e., those closest to the axis of rotation, only the Al_2_O_3_ phase is observed. These areas are more expansive for composites cast at higher speeds. The further from the sample’s axis, the wider the zones are in the metallic phase. In the areas at the outer edge of composites, we observe smaller particles of the metallic phase. This area is the zone that was first created in the molding process by suction of the liquid by capillary forces acting in the micropores of the gypsum mold. For each of the series of composites, the width of the zone with the most significant proportion of the metallic phase increases with the increase in the proportion of the metallic phase. Comparing the microstructures of composites with the exact contents of the metallic phase, we can conclude that the higher casting speed favors a greater concentration of the metallic phase particles. The observations revealed a good density of the composites, except for the central areas in the microstructure of the B_10 and B_15 composites, where we can see larger pores. High porosity was also observed in the A_10 composite and the A_15 sample. No microcracks were observed in the microstructure of the composites of both series, which could appear at the metal-ceramic interface due to differences in thermal expansion and significantly weaken the strength of the material.

In the central zones of the composite microstructure, where the accumulation of the metallic phase was the highest, it was observed that more red metallic phases appeared from the side of the outer edges and more of the light-yellow phase closer to the inner edge. In these zones, the distribution of the metallic phase was affected by the centrifugal force, which depends on the mass of the particle it affects and the centrifugal acceleration. Since copper is denser than chromium, the red metallic phase is likely copper, and the light-yellow metallic phase is chromium. Figure 8 shows the microstructure of composites in cross-section. This phenomenon is best visible for the B_15 composite.

**Figure 9:**
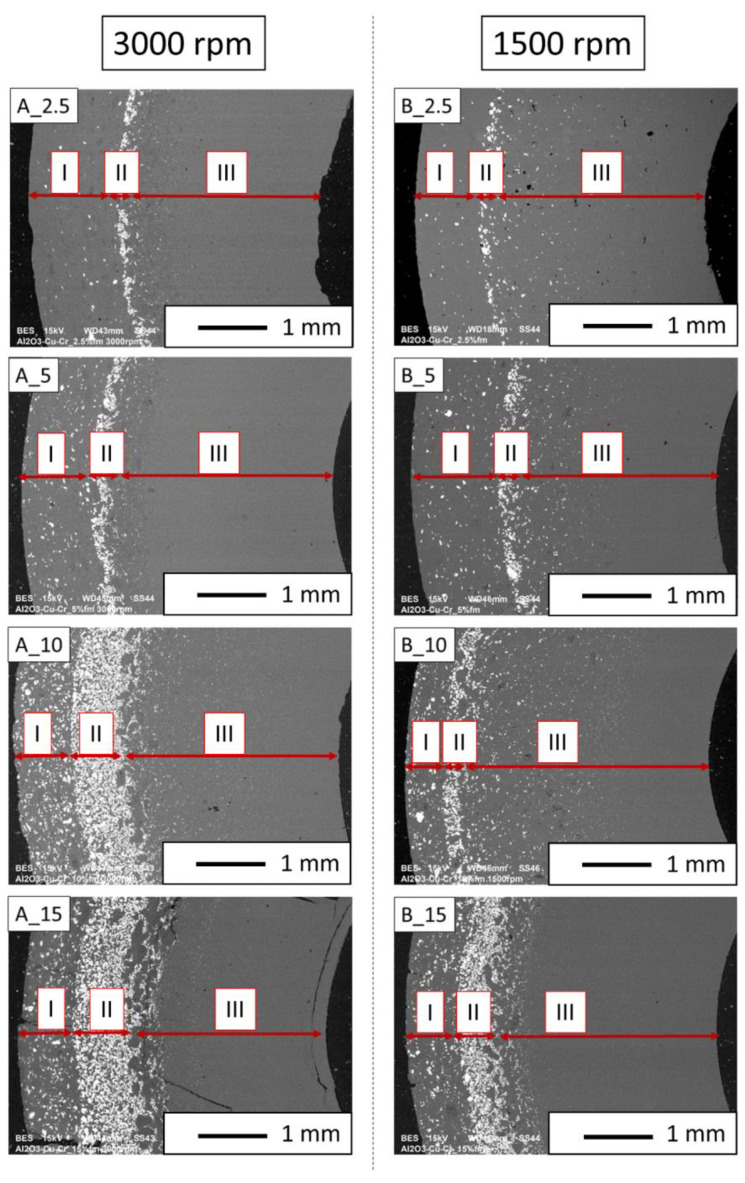
SEM images of the microstructure of composites on a cross-section.

Then, observations were made using a scanning electron microscope for a more accurate analysis of the obtained microstructures. Based on SEM images of cross-sections of composites, the microstructure was divided into three zones (Figure 9) depending on the degree of agglomeration of the metallic phase. The zone marked as I is the area closest to the outer edge of the sample. It is formed immediately after pouring the slip into the mold and before starting the centrifuge. The metallic particles are evenly distributed, and their proportion should be the same as the average proportion in the slip. Zone II is where the greatest concentration of the metallic phase occurred. About the entire cross-section, the concentration of metallic particles is the highest in this zone. The size distribution of the metallic phase areas in this zone results from the centrifugal force. In the initial phase of the casting process, when there is a large amount of liquid in the suspension, the particles of the metallic phase, having a higher density than ceramic particles, move faster toward the outer surface of the mold under the action of the centrifugal force (in the direction of the centrifugal force). The greater the centrifugal force, the larger the particle’s diameter. Over time, as the solvent is removed, the viscosity of the slurry increases. This phenomenon proceeds from the outer surface (contact with the mold) towards the axis. Moving metallic particles hit an obstacle in the form of a boundary between compacted and uncompacted mass (with a viscosity that allows particles to move). Thus, Zone II is formed when the moving particles stop at the boundary of Zone I. The zone marked as III is the area from the inner edge of the sample to the area where the concentration of metallic particles is the greatest. In this area, there are no particles of the metallic phase at the inner edge. It can be seen that their number and size increase gradually in the direction from the axis of the sample to the outer edge.

**Figure 10:**
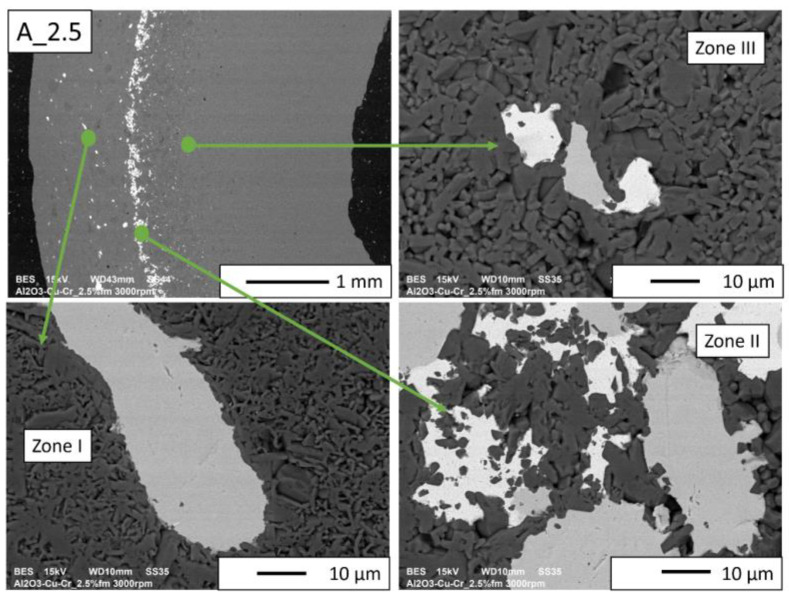
SEM image of the A_2.5 composite microstructure.

**Figure 11:**
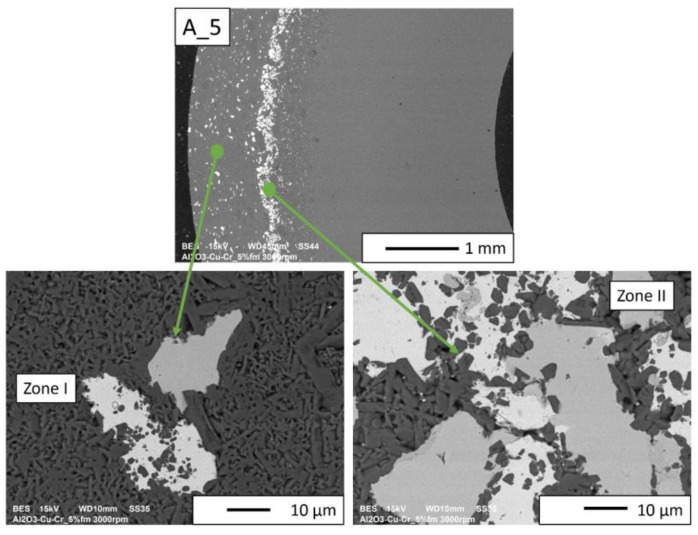
SEM image of the A_5 composite microstructure.

**Figure 12:**
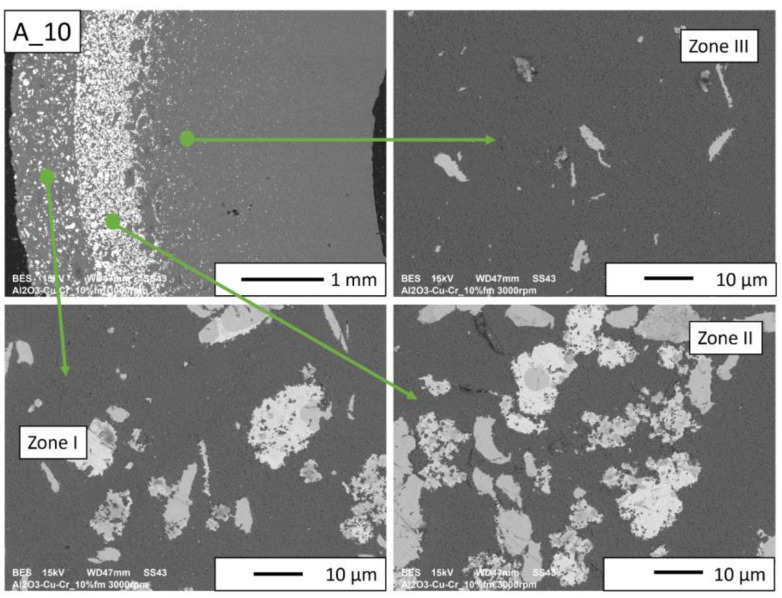
SEM image of the A_10 composite microstructure.

**Figure 13:**
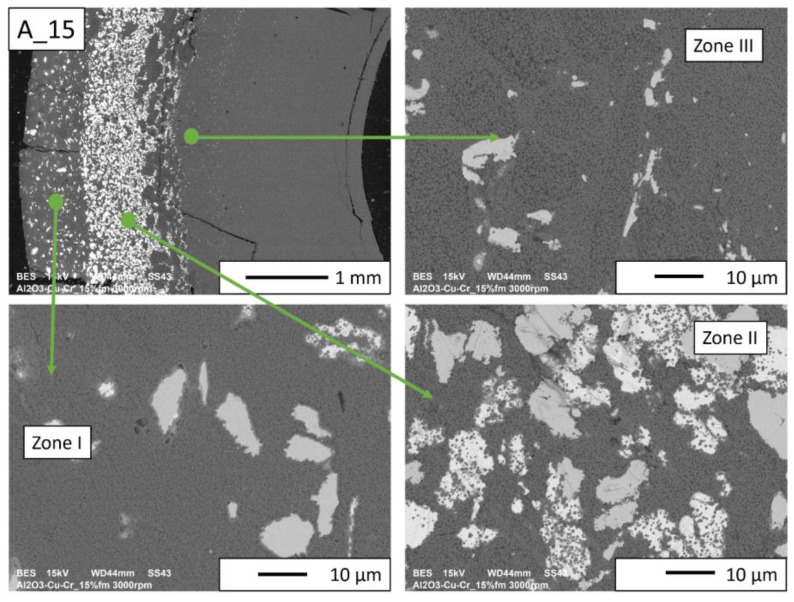
SEM image of the A_15 composite microstructure.

According to the SEM images of the microstructure of the composites, we can observe that two metallic phases are visible in the matrix. The lighter phase is likely copper, which has a higher atomic number, and the darker phase is chromium. This was confirmed in further EDS studies. According to the SEM observations of the microstructure of the A series composites (Figure 10, Figure 11, Figure 12 and Figure 13), the particles of the metallic phase in Zones I and III are separated. In Zone II, a tendency of copper to surround chromium particles was observed, increasing with the increase of the content of the metallic phase. This proves the good wettability of chromium by liquid copper.

The SEM images of the A_2.5 and A_5 composites (Figure 10 and Figure 11, respectively) revealed that all distinguished gradient zones achieved a good connection between the reinforcement and the matrix. There are no delaminations and porosities at the phase separation boundary. It can be observed that the copper in the sintering process penetrated between the ceramic particles. This is particularly noticeable in Zone II, where the highest accumulation of the metallic phase occurred.

In the case of A_10 and A_15 composites (Figure 12 and Figure 13, respectively), there are no porosities and delaminations at the phase separation boundaries. In Zone II, spherical chromium particles are visible inside the copper regions. Ceramic particles are visible in the copper areas. This proves that a suitable penetration of liquid copper between the particles was obtained. Based on the images of the microstructure of these composites, it can be seen that the increase in the chromium content improved the wettability of the matrix by liquid copper. The presence of Cr particles, well wetted by Cu, makes the liquid phase better distributed inside the sample. The structure closest to the percolation one was obtained for Zone II of the A_15 composite.

**Figure 14:**
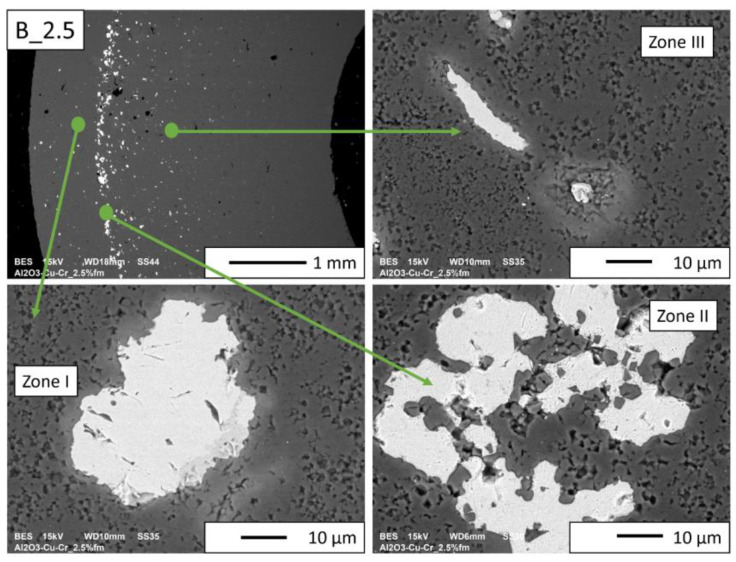
SEM image of the B_2.5 composite microstructure.

**Figure 15:**
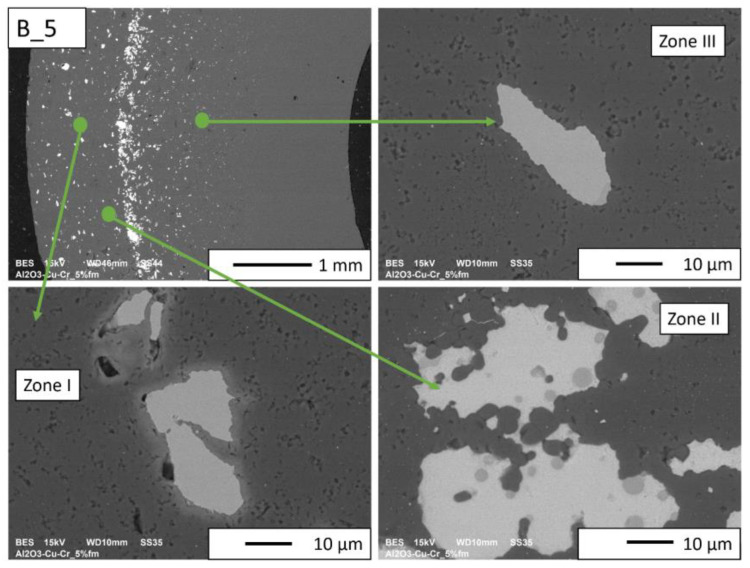
SEM image of the B_5 composite microstructure.

**Figure 16:**
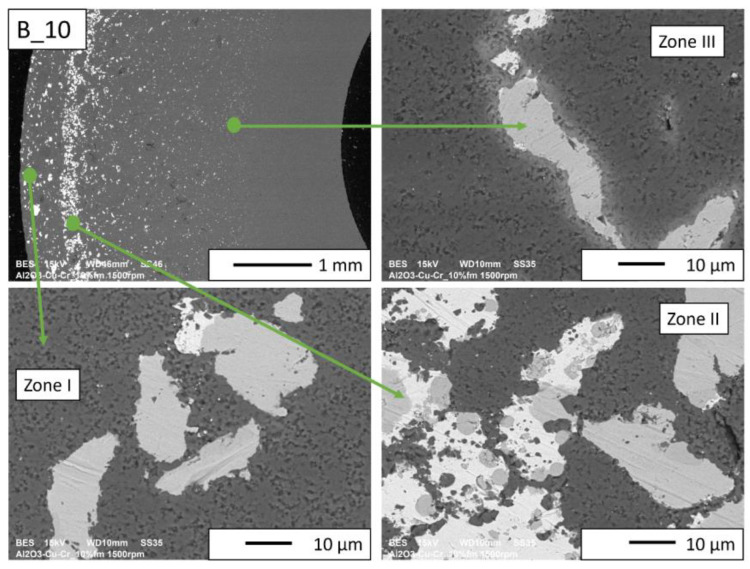
SEM image of the B_10 composite microstructure.

**Figure 17:**
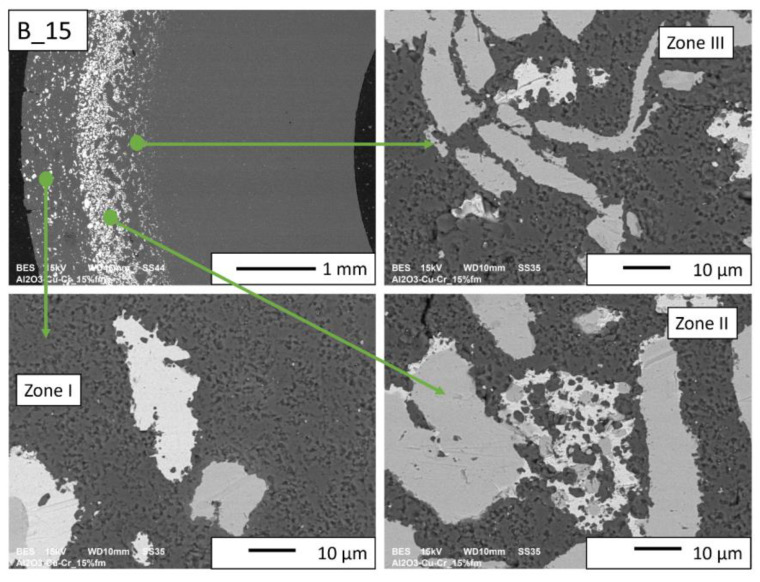
SEM image of the B_15 composite microstructure.

SEM observations for the B series composites (Figure 14, Figure 15, Figure 16 and Figure 17) revealed that the particles of the individual metallic phases in Zones I and III are mostly separated. In Zone II, the most rich in the metallic phase, chromium particles of spherical shape were observed inside the copper regions. This shape most likely results from the partial dissolution of chromium in liquid copper, which leads to a decrease in the free energy of the particles through their coagulation. Such a microstructure proves the good wettability of chromium particles by liquid copper, which flowed around chromium during the sintering process.

In the case of B_2.5 composites and in Zones I and III of the B_5 composite (Figure 14 and Figure 15), pores can be observed at the boundaries of the separation of the metallic phase and Al_2_O_3_.

No porosity was observed at the interface between the metallic phase and the matrix for the B_10 and B_15 composites (Figure 16 and Figure 17, respectively). In addition, it was observed that in Zone II of these samples, copper is located between small ceramic particles, which indicates an improvement in the wettability of the matrix by copper with the increase in the proportion of the metallic phase. At the sintering temperature (1400 °C), approximately 8% Cr is dissolved in copper by weight. A solution can only form where the copper and chromium particles are in contact. The probability of contact between the particles of both metals increases with the increase in the proportion of the metallic phase. In the gradient sample, it is the largest in Zone II. Most likely, the chromium content not exceeding 5%_vol_ is insufficient for the reaction between the copper and chromium particles to occur.

According to the microstructure analysis, an increase in the chromium content improves the wettability of the ceramic by liquid copper. Higher casting speed also favors better wettability because it leads to a greater concentration of particles of both metals in Zone II.

### 3.1. Chemical Composition Analysis

**Figure 18:**
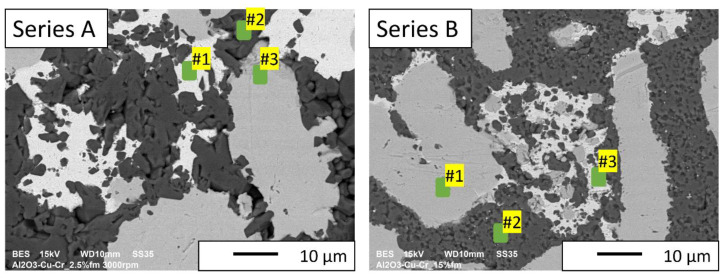
SEM images with marked points where the EDS analysis was performed.

Based on the selected results of the chemical composition analysis, it was found that Cu, Cr, Al, and O_2_ were present in the microstructure of the A and B series composites (Table 6). The tests confirmed earlier assumptions that the areas can be classified as follows: the lighter metallic phase is copper, the darker is chromium, and the darkest areas are aluminum oxide (Figure 18).

### 3.2. Image Analysis

In the next step, the focus was on the results of the stereological analysis of the metallic phase areas in each of the three designated zones of the obtained microstructure gradient.

**Figure 19:**
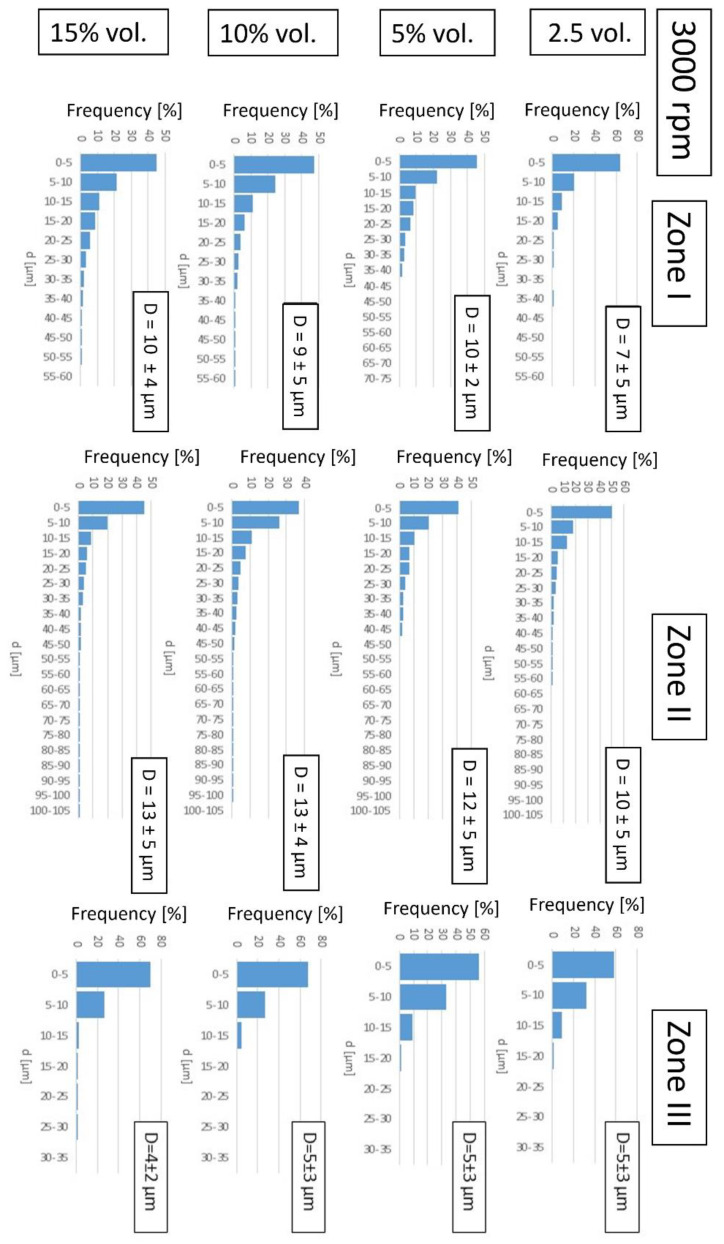
Histograms of the size of the metallic phase agglomerates of the composites produced for each zone along with their average size—D for a series of samples produced at 3000 rpm.

**Figure 20:**
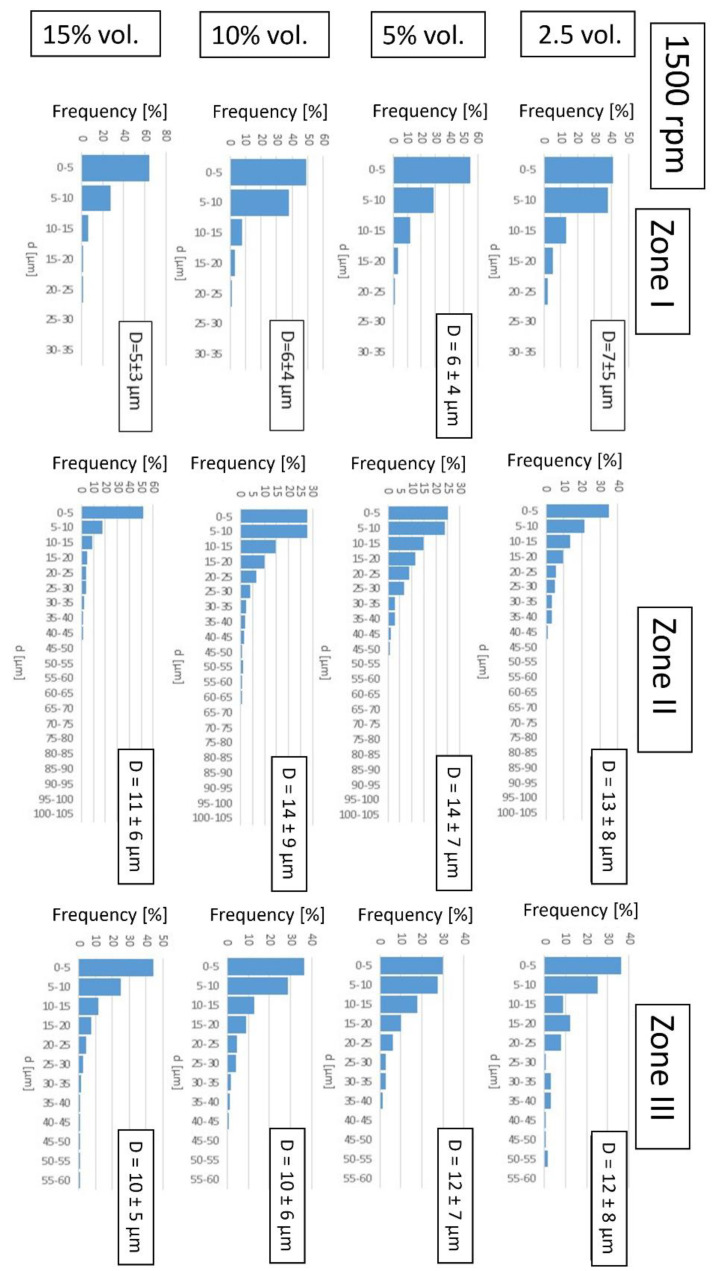
Histograms of the size of the metallic phase agglomerates of the composites produced for each zone along with their average size—D for a series of samples produced at 1500 rpm.

According to the stereological analysis, the most numerous fractions in all zones of both series are particles with a diameter below 10 µm (Figure 19 and Figure 20). Comparing these data with the results of the DLS analysis of metal powders, according to which the most numerous fractions of metal powders are particles with a diameter of about 10 µm, it can be concluded that the particles were broken down into smaller ones in the casting process. Comparing the average particle size in each zone for composites of both series with the same content of the metallic phase, we can see that they are slightly lower for composites cast at a higher speed. It can, therefore, be assumed that the powder particles are agglomerated as they are in their original state. These agglomerates were not completely separated during the DLS measurement but were broken up during slip preparation and centrifugal casting.

In Zone I, the particle size range increases with the increase in the content of the metallic phase. With the increase in the content of the metallic phase, the number of particles in this area increases, and thus, the probability of finding a certain number of particles in the immediate vicinity increases. These particles fuse into more significant areas of the metallic phase during sintering.

In Zone II, the size range of metallic areas increases with the increase in the proportion of the metallic phase. An increase in the frequency of occurrence of more prominent areas of the metallic phase was also observed at the expense of smaller ones, with an increase in the content of the metallic phase to 10%_vol_. This is due to the high concentration of particles in this zone leading to their merging.

For the content of the metallic phase of 15%_vol_, the frequency of occurrence of larger areas is slightly lower, which may be related to the most considerable loss of the metallic phase during sintering.

In Zone III, at the rotational speed of 1500 rpm, the size ranges of metallic areas are the same, which can be explained by the fact that a small share of particles in this zone are isolated and do not merge into larger areas. At a casting speed of 3000 rpm, in a sample containing 15% vol. of the metallic phase, a fraction of metallic areas in the range of 25–30 µm was observed, but these were single counts.

Table 7 summarizes the share of the metallic phase in individual zones of the composites produced. It was found that the content of the metallic phase in individual zones increases with the increase in the proportion of the metallic phase in the slip used to form the samples and the casting speed. The largest share of the metallic phase was determined for Zone II and the smallest for Zone III. The proportion of metallic particles in Zone I is lower than assumed (such as in the slip). This may result from segregation when the mass is compacted, or copper flows out during sintering. The results show that by centrifugal casting, it was possible to obtain a strong differentiation of the composite structure along the radius of the sample. This certainly substantially impacts the mechanical properties of the obtained materials, which should be investigated in the next stage of work.

## 4. Summary

A relatively high relative density characterizes the produced ceramic–metal composites. In the macroscopic assessment, the best quality composites are the samples with the lowest content of the metallic phase, amounting to 2.5%_vol_. These composites lack defects in the form of cracks, porosity, and depletion in the metallic phase, resulting from copper leakage during the sintering process. Introducing a more significant amount of the metallic phase into the matrix resulted in numerous cracks and the outflow of copper onto the composite surfaces. According to the results obtained during hydrostatic weighing, the relative density of the A series composites (cast at 3000 rpm) ranges from 80.35 ± 0.43% to 95.7 ± 0.48%. In the B series (cast at 1500 rpm), the obtained relative densities range from 93.68 ± 1.09% to 97.74 ± 0.31%. Therefore, all produced composites are characterized by a high relative density, while using a lower casting speed was conducive to obtaining a higher density. Observations of the microstructure of the cross-sections of the produced composites revealed a gradient of the distribution of the metallic phase particles, in which, based on the proportion and distribution of the metallic phase, three zones with different values of these parameters can be distinguished. SEM observations of the microstructure of composites revealed that in the sintering process, the proportion of interfacial boundaries increased with the increase in the content of the metallic phase. The resulting Cr solution in Cu more effectively filled the spaces between the ceramic particles. This may be related to the improved wettability of alumina with liquid copper by chromium, as indicated by literature data. Comparing the microstructure of the composites of both series with the exact content of the metallic phase, it was noticed that a better connection at the interface of ceramic and metal phases was obtained for higher casting speed. According to the stereological analysis, the most numerous fractions in all zones of the produced composites are metallic areas with a diameter below 10 µm. The share of the metallic phase in each zone increased with the metallic phase content in the composite and the casting speed. A more significant proportion of the metallic phase in each zone resulted in forming a larger area of Cu-Cr interfaces and, thus, creating a more considerable amount of Cr solution in liquid copper, which probably wet the ceramics better than liquid copper. This explains the better connection between the matrix and the reinforcement for a higher casting speed.

## 5. Conclusions

Based on the conducted research, the following conclusions can be drawn:

1. The method of centrifugal slip casting is a technology that allows the production of ceramic–metal composites with a microstructure gradient and high density.
2. Adding chromium to the Al_2_O_3_-Cu system improves the connection between the ceramic and metal particles, probably by reducing the contact angle at the interface between the ceramic and metallic phases during sintering. However, this did not completely eliminate the phenomenon of copper flowing onto the material’s surface.
3. An increase in the content of the metallic phase above 5%vol leads to a large proportion of the metallic phase in the central part of the cross-section of the composites (in Zone II).
4. Higher casting speed was conducive to obtaining a better connection at the interface of ceramics and metal.

## Figures and Tables

**Table 1:** Specification of the powders used in the research based on the manufacturers’ data.

Material	Average Particle Size	Density	Purity
α-Al_2_O_3_	100 ± 25 nm	3.98 g/cm^3^	99.99%
Cu	<150 µm	8.94 g/cm^3^	99.98%
Cr	<50 µm	7.14 g/cm^3^	99.99%

**Table 2:** Calculated theoretical densities of the produced composites depending on the content of the metallic phase based on the rule of mixtures.

Volume Fraction of the Metallic Phase in the Composite (%)	ρ_t_ (g/cm^3^)
2.5	4.06
5	4.16
10	4.37
15	4.57

**Table 3:** Samples produced by centrifugal casting.

Composite Al_2_O_3_-Cu-Cr	Casting Speed (rpm)	Metallic Phase Content (%_vol_)	Sample Designation
Series A	3000	2.5	A_2.5
		5	A_5
		10	A_10
		15	A_15
Series B	1500	2.5	B_2.5
		5	B_5
		10	B_10
		15	B_15

**Table 4:** The results of pycnometric and BET tests and the d_BET_ values of powders calculated on their basis.

Material	ρ (g/cm^3^)	S_(BET)_ (m^2^/g)	d_BET_
Al_2_O_3_	3.8949 ± 0.0427	10.85 ± 0.07	142 ± 2 nm
Cu	9.0386 ± 0.0237	0.09 ± <0.01	7.38 ± 0.85 μm
Cr	7.1277 ± 0.0447	0.67 ± 0.02	1.256 ± 0.045 μm

**Table 5:** Selected physical properties determined by the Archimedes method.

Casting Speed	Al_2_O_3_-Cu-Cr Composite	Relative Density ρ_w_ (%)	Open Porosity P_o_ (%)	Water Absorption N (%)
3000 rpm	A_2.5	93.56 ± 1.34	4.11 ± 1.49	1.08 ± 0.41
	A_5	93.06 ± 0.32	0.63 ± 0.12	0.16 ± 0.03
	A_10	95.70 ± 0.48	0.72 ± 0.14	0.17 ± 0.03
	A_15	80.35 ± 0.43	9.62 ± 1.92	2.62 ± 0.53
1500 rpm	B_2.5	97.74 ± 0.31	0.21 ± 0.07	0.05 ± 0.02
	B_5	96.80 ± 0.35	0.54 ± 0.4	0.13 ± 0.1
	B_10	93.68 ± 1.09	0.56 ± 0.36	0.14 ± 0.09
	B_15	95.81 ± 0.2	0.77 ± 0.12	0.18 ± 0.03

**Table 6:** Results of the EDS analysis of composites.

Point	Amount of the Element [%wt]			
	Al	O	Cu	Cr
Series A #1	-	0.8 ± 0.1	97.6 ± 0.1	1.5 ± 0.1
Series A #2	48.3 ± 0.1	43.8 ± 0.1	-	8.0 ± 0.1
Series A #3	-	-	0.9 ± 0.2	99.1 ± 0.2
Series B #1	-	-	-	100
Series B #2	51.5 ± 0.2	44.3 ± 0.2	-	4.2 ± 0.1
Series B #3	-	0.8 ± 0.1	97.0 ± 0.1	2.2 ± 0.1

**Table 7:** Participation of the metallic phase in individual gradient zones of the produced composites.

Metallic Phase Content	Metallic Phase Content (%)					
	3000 rpm			1500 rpm		
	Zone I	Zone II	Zone III	Zone I	Zone II	Zone III
2.5%_obj_	0.66	10.92	0.40	0.64	8.93	0.14
5%_obj_	3.41	23.86	0.68	0.67	10.92	0.56
10%_obj_	8.47	45.24	1.09	4.47	28.93	0.80
15%_obj_	9.84	47.38	1.69	5.43	42.17	0.97

## Data Availability

Data sharing not applicable.
